# A novel intrauterine satellite transmitter to identify parturition in large sharks

**DOI:** 10.1126/sciadv.add6340

**Published:** 2023-03-01

**Authors:** James A. Sulikowski, Neil Hammerschlag

**Affiliations:** ^1^ School of Mathematical and Natural Sciences, Arizona State University, Glendale, AZ 85306, USA.; ^2^ Rosenstiel School of Marine and Atmospheric Science, University of Miami, Miami, FL 33149, USA.

## Abstract

Determining where and when animals give birth is critical for establishing effective conservation management that protects vulnerable life stages (e.g., pregnant females and newborns) and places (e.g., nursery grounds). To date, this information has been elusive in the case of highly migratory sharks in the wild. Here, we report on the deployment a of novel intrauterine satellite tag implanted in two highly mobile apex predators, the tiger shark (*Galeocerdo cuvier*) and the scalloped hammerhead (*Sphyrna lewini*), that remotely documented the location and timing of birth by a highly migratory oceanic animal in the wild. This novel technology will be especially valuable for the protection of threatened and endangered shark species, where protection of pupping and nursery grounds is a conservation priority.

## Teaser

An intrauterine satellite tag implanted in two wild shark species remotely documented the location and timing of birth.

## INTRODUCTION

Understanding the reproductive biology of large marine predators, such as sharks, is important for establishing effective conservation. Specifically, data on maturity state, gestation period, breeding frequency, and fecundity are needed for generating accurate population models and determining when a population is overexploited (*1*, *2*). Moreover, understanding these reproductive characteristics is necessary for establishing effective site-based management strategies, such as marine protected areas or time/area closures to safeguard key life-history stages (*3*). However, reproductive data on fishes are traditionally derived from sacrificing specimens and inspecting reproductive organs (*4*, *5*). In addition, the scale and often remote movements of marine predators make identifying their critical reproductive sites especially challenging (*6*). Delineating this information is particularly important for sharks because their general life-history characteristics (slow growth, low fecundity, and long-lived) make them highly susceptible to both anthropogenic and environmental change (*7*, *8*).

Although certain adult shark migrations have been linked to postulated reproductive events [e.g., mating; (*9*)], empirical data to support these movements are rare and have only been observed in relatively lower trophic level shark species that exhibit low mobility (*10*–*13*). The delineation of parturition sites or proposed nursery grounds (*14*) has typically been inferred from the capture of gravid individuals or neonate sharks (*15*, *16*) or based on parental genotype reconstruction in nearshore areas (*17*–*19*). While there are limited data revealing that adults of some shark species return to their respective parturition sites to give birth, the timing and exact location of when and where these events occur in highly mobile species remain speculative at best, as gestation grounds may differ from parturition or nursery grounds and young-of-the-year sharks have the capacity to move relatively long distances immediately after birth (*20*). Until new techniques or technologies are developed, the exact timing and locations of parturition will remain unknown, especially in the case of highly mobile oceanic species. Here, we report on the deployment and results of a novel intrauterine satellite tag implanted in two highly mobile apex predators, the tiger shark (*Galeocerdo cuvier*) and scalloped hammerhead (*Sphyrna lewini*), that successfully documented the location and timing of their parturition along the southeastern coast of the United States.

Used in terrestrial systems, intrauterine tags, or vaginal implant transmitters (VITs), are radio transmitters inserted into the vaginal canal of a female ungulate, and sealed with a flexible material, to be expelled at parturition (*21*). Since the 1980s, VITs have been used to identify the timing and location of parturition sites of ungulates for estimating juvenile survival (*21*). However, to date, this tool had never been applied to marine systems, likely owing to the inherent technical and logistical challenges of working in open aquatic systems and with large marine megafauna. However, recent technological advances in aquatic animal tracking, including tag miniaturization and sensor development, have made it possible to create a waterproof, satellite-linked VIT. In addition, unlike mammals, during gestation in sharks, the entrance to the uterus remains semi-permeable to allow for water exchange (*22*). Accordingly, with the aid of a specialized applicator and guided by in situ ultrasonography, it is feasible in gravid sharks to implant a transmitter into the cloacal opening, through the urogenital canal, into the uterus, and deposit it among developing embryos where the VIT would remain until parturition when it is expelled with the young (*22*, *23*).

**Fig. 1.**
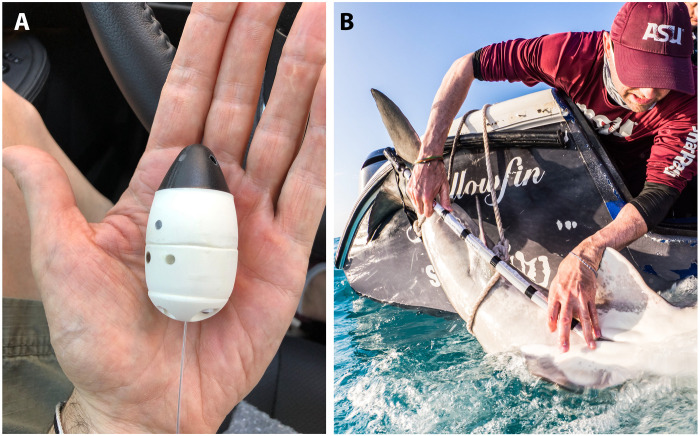
Image of BAT and applicator. (**A**) Representative image of the BAT. The egg-shaped device measures 6 cm in length by 2.5 cm in width and weighs 42 g in the air. (**B**) Insertion of the BAT via a specialized applicator into the cloacal opening of a gravid female tiger shark on 15 December 2019, also guided by simultaneous ultrasonography. Photo credit: T. Houppermans.

Beginning in spring of 2017, we worked with Lotek Inc. (Newfoundland, CA) to develop a waterproof satellite-linked VIT, hereafter termed the birth-alert-tag (BAT). The BATs were designed to be egg-shaped, 6 cm in length by 2.5 cm in width, with a flexible antenna, and weigh just 42 g in the air (Fig. 1A). The shape and size were chosen to facilitate entry into the female shark’s uterus (*23*) and to remain inconspicuous among the developing embryos. The electronics were encased in an egg-shaped buoyant synthetic foam covered with a biologically inert sealant that, upon expulsion at birth, would float to the surface. The BATs were equipped with a wet/dry sensor that allowed the unit to go into a standby mode (on) once it detected the aqueous environment of the shark’s uterus. Here, the tag remained on (but not transmitting) until the wet/dry sensor subsequently detected a dry reading, which would only occur once the tag was expelled from the uterus and floated to the ocean surface, exposing the tag’s antennae skyward. At this point, the tag would switch into a transmitting mode, sending messages at 15-s intervals to be detected by ARGOS (Advanced Research and Global Observation Satellites) (*24*) until its battery expired (15 days). We worked with The Custom Shop (Amherst, MA) in the development of a specialized applicator (Fig. 1B) to insert the BAT through the cloacal opening into the uterus.

Our ability to determine the success of BAT deployments in tiger sharks and scalloped hammerheads benefited from known information on the reproductive biology of these species. Specifically, the seasonal timing of parturition for tiger sharks and scalloped hammerheads has previously been inferred from the capture of young of the year along the United States (tiger sharks: 25; 15; scalloped hammerheads: 26). Accordingly, this allowed us to compare the seasonal timing of birth identified by the BATs with known timing of parturition in both species. In addition, embryo size at various stages of gestation is known for both tiger sharks and scalloped hammerheads based on euthanized specimens (*27*–*29*). Therefore, by measuring the size of developing embryos via ultrasonography at the time of BAT implantation and comparing it to the known size at various stages of development, including birth, we could predict the duration to parturition and thus the period of expected BAT tag release as another form of validation.

## RESULTS AND DISCUSSION

**Fig. 2.**
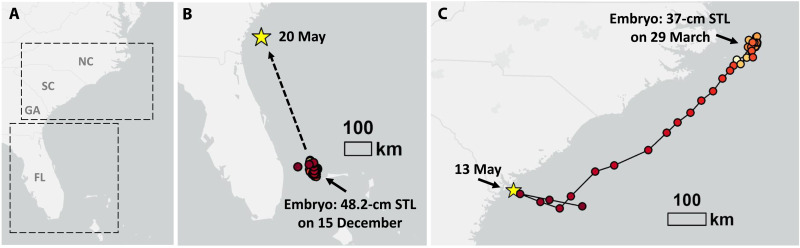
Parturition locations for tiger and scalloped hammerhead sharks as indicated by the BAT release. (**A**) Eastern seaboard of the United States and Northern Bahamas, with upper and lower boxes identifying tracking areas of the gravid tiger shark (bottom) and scalloped hammerhead (top), further detailed in (B) and (C), respectively. Florida (FL), Georgia (GA), South Carolina (CA), and North Carolina (NC) are identified for spatial reference. (**B**) Capture and tagging location of the gravid female tiger shark at the gestation site on 15 December 2019, where ultrasound imaging revealed an estimated embryo size of 48.2-cm stretched TL (STL). The yellow star represents the location of BAT release 157 days after initial capture in a known tiger shark nursery area. (**C**) Capture, tagging, and parturition location for the gravid female scalloped hammerhead. The shark was captured and tagged on 29 March 2022, where the ultrasound revealed an estimated embryo size of 37.0-cm STL. After spending 31 days in the vicinity of the tagging site, the shark made a directed, rapid, southward movement along the edge of the continental shelf for 2 weeks, where it then abruptly changed direction, veering west inshore, following which the BAT was expulsed in a known scalloped hammerhead nursery area, 46 days after initial capture, after which the female then reverted back eastward to offshore waters along the edge of the continental shelf.

On 15 December 2019, a female tiger shark (ID#147602) measuring 338 cm in total length (TL) was captured on the little Bahama Bank, Bahamas (26.90471-79.06207; Fig. 2, A and B), a putative gestation ground in the western Atlantic for this species (*30*). The shark was slowly brought to and secured alongside the boat, rolled over on her back, and put into a state of tonic immobility, such that she was partially submerged in the water, with her ventral surface facing skyward. Ultrasonography was performed using an Ibex EVO II portable ultrasound (EI Medical Imaging) with a CL3E 2.5- to 5-MHz transducer to identify the shark was gravid. A BAT was then manually inserted using the specialized applicator into the cloacal opening, through the urogenital canal, and into the left uterus. To track the location of the female post-release, a fin-mounted satellite tag (SPOT5, Wildlife Computers) was affixed to her dorsal fin, providing satellite-linked positions whenever the fin surfaced and sufficient transmissions where received by ARGOS satellites.

**Fig. 3.**
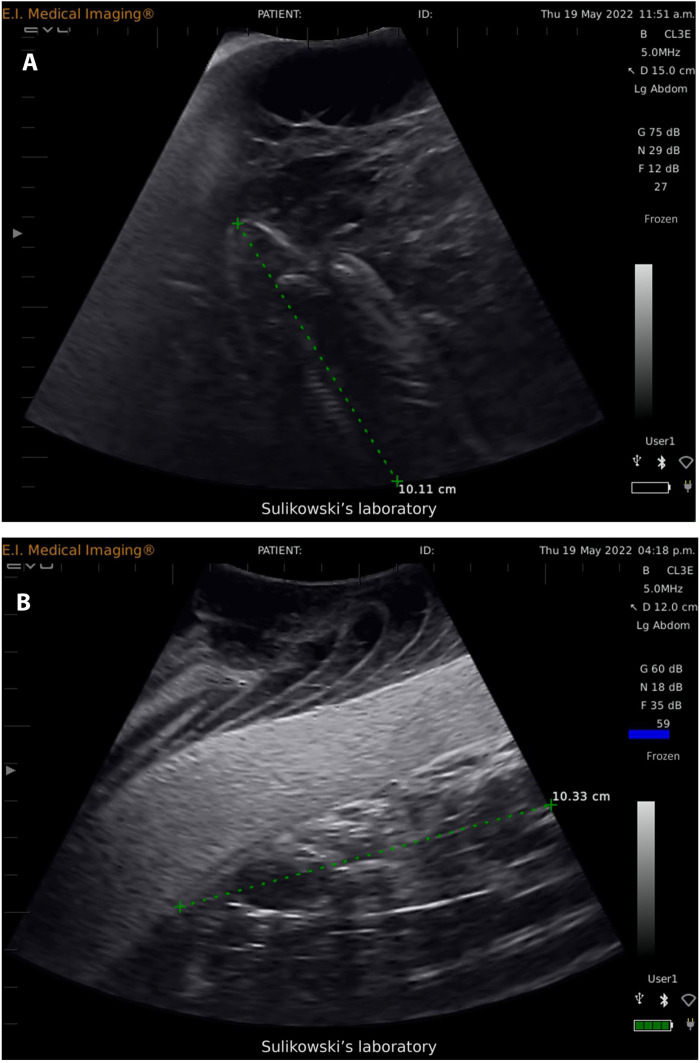
Ultrasound images of embryonic tiger and scalloped hammer head sharks at time of BAT deployment. Representative ultrasound images of an embryonic tiger shark [(**A**); tip of rostrum to behind last gill arch] and scalloped hammerhead [(**B**); tip of rostrum to anterior of second dorsal fin] from the gravid females tracked with the BAT. Green dotted lines represent lengths in centimeters measured from proprietary software (10.11 cm for the tiger shark embryo and 10.33 cm for the scalloped hammerhead embryo) preinstalled on an Ibex EVO II ultrasound. Embryonic shark STLs of 48.2 cm (tiger shark) and 37.0 cm (scalloped hammerhead) were estimated by using measurements obtained from the ultrasound and applying the measurements to mathematical conversion formulae in (*25*) and (*31*, *32*) (see manuscript text for details).

Analysis of collected images from multiple tiger shark embryos and video loops (via proprietary software preinstalled on the Ibex EVO II ultrasound) indicated that mean tiger shark embryo size was approximately 35-cm precaudal length (PCL; Fig. 3A). The PCL was converted to stretched TL (STL), measuring 48.2 cm, according to published length-to-length relationships (*31*). On the basis of this size, we estimated the gravid shark to be two-thirds through gestation, with parturition to occur between May and July 2020, coinciding with the occurrence of neonates in the western North Atlantic (*25*, *15*). After release, the tiger shark’s fin-mounted tag indicated that the shark remained within the gestation site, until at least 7 February 2020, when the tag transmitted its last position due to battery failure. On May 20 at approximately 1:28 a.m.GMT time, after 157 days in utero, the BAT was detected by the ARGOS satellite system at location 31.29051, −80.8001 (Fig 2B), approximately 40 km off the coast of Southern Georgia, USA, in an area that was approximately 40-m deep. This area appeared to be without structure and consisting of sandy bottom. At the time of detection, there had been satellites overhead the BAT evacuation location from 20:30 on 19 May to 05:00 on 20 May, indicating that there was ample satellite coverage to detect the BAT if it had been evacuated earlier. BAT transmissions were strong [ranging between 1 and 3 location class (LC)]. The BAT transmitted 27 times within the first 24 hours after release and continued transmitting for 11 days within this location before the battery expired.

On 29 March 2022, a female scalloped hammerhead (ID#228328), measuring 304 cm in TL, was captured off the coast Cape Hatteras, North Carolina (35.24572, -75.31022; Fig. 2C). The scalloped hammerhead was handled, measured, ultrasonography performed, implanted with a BAT (left uterus), and equipped with a fin-mounted satellite tag in the same manner as described above for tiger sharks. Analysis of collected images of multiple embryos for the scalloped hammerhead was converted from PCL to STL (*31*) and indicated that the mean embryo size was 37.0-cm STL (Fig. 3B), suggesting that parturition would most likely occur between May and June 2022, coinciding with the timing of parturition predicted for this species based on data from previously euthanized specimens (*29*) and the occurrence of young of the year from past studies in the region (*26*).

Unlike the movement data associated with the tiger shark, the scalloped hammerhead’s fin-mounted tag produced daily transmissions through to parturition. Following tagging and release, the shark remained offshore, within the vicinity of the tagging area for 31 days (Fig. 2, A and C). On 29 April, the shark initiated a southward directional change in movement, traveling 420 km along the edge of the continental slope, until 12 May, upon where the female shark abruptly turned west, making a directed movement inshore (Fig. 2C). On 13 May 2022, at 09:22:37 GMT time, after 46 days in utero, the BAT transmitted at 32.14039, -80.54074 (Fig. 2C), a location that was approximately 12 km off the coast of Hilton Head, South Carolina, USA, in an area with a water depth of approximately 20 m. This area appeared to be with live bottom and artificial structure and is a known coastal nursery area for this species along the U.S. Atlantic east coast (*26*). Ample satellite coverage was overhead at the time of the BAT evacuation. In addition, at 9:26:53 a.m. GMT, the fin-mounted tag transmitted a location of 32.11446, -80.58654, less than a kilometer from the first BAT transmission. The expelled BAT was subsequently detected 16 times, from first detection through 16:39:40 GMT, and continued transmitting for 11 days within this area until the battery expired. After parturition, the shark immediately left the coastal parturition site and moved rapidly offshore again toward the shelf break (Fig. 2, A and C).

While we understand that we cannot fully rule out the possibility the BAT was prematurely expelled before birth, high confidence that the BAT was successfully released at parturition in both species comes from four primary lines of complementary evidence. First, premature evacuation would have been expected to occur within hours of implantation (*33*); a duration well exceeded here. Second, the timing of BAT evacuation occurred during the known window of parturition for both species in the region based on the occurrence of neonates (25; 15). Third, the timing to BAT expulsion was consistent with the time that it would take the embryos measured in utero to reach their known range in size at birth (61- to 85-cm STL in tiger sharks; 26, 15, 29; and 38.5- to 50-cm TL for scalloped hammerheads; 26) within the region. Last, the tracked movement behaviors of both species indicated parturition where the BAT release site. In the tiger shark, the BAT remained in utero during the entirety of the shark remained at the gestation ground, only releasing months later once the shark had departed for birthing grounds during the birthing season. In the case of the hammerhead, this species is semi-pelagic, occupying offshore waters as adults, although nursery grounds are known to occur inshore. Here, this shark remained offshore, except for the directed, rapid migration to the inshore location where the BAT released, after which the shark immediately returned to offshore waters. Thus, on the basis of these data, we believe both sharks carried the BAT until parturition. In addition to identifying the location and timing of parturition, BATs have the potential to provide insights in neonate dispersal capabilities if locations of captured or observed young of the year differ from parturition sites identified by BATs. We believe that this tool will be particularly valuable for identifying the parturition location and timing of threatened and endangered species where protection of pupping and nursery grounds is critical. It is our hope that when BATs become commercially available, groups of scientists across disciplines will collaborate to use this tool. Furthermore, there is interest from the manufacturer in reducing the size of this technology, which we anticipate will permit its use in a wider array of elasmobranch species.

## METHODS

### Capture and handling

The female tiger shark was captured, sampled, and tagged on the northwest edge of little Bahama Bank, Bahamas, at a site nicknamed “Tiger Beach” (26.86°N, 79.04°W), a putative gestation ground for the species (*31*). The female scalloped hammerhead was captured, sampled, and tagged approximately 20 km northeast of Cape Hatteras, North Carolina (35.24572 -75.31022). The tiger shark was captured using standardized circle-hook drumlines, while the scalloped hammerhead was captured using a standard bluefin tuna class rod and reel. Once hooked, each shark was slowly brought alongside the research vessel, where it was carefully restrained. For each individual, sex was recorded and STL was measured to the nearest centimeters over a straight line along the axis of the body using a tape measure.

### Ultrasound and BAT

Ultrasonography was performed following Sulikowski *et al*. (*30*). Briefly, an Ibex EVO II portable ultrasound (EI Medical Imaging) with a 60-mm curved linear array 2.5- to 5-MHz transducer (model 290470) capable of a 24-cm scan depth was used to obtain images of the reproductive tract of each female. Scanning was performed on the ventral surface from the pectoral to the pelvic fin in both a transverse and longitudinal orientation to obtain cross-sectional and lengthwise images, respectively. Depth of the scan ranged from 12 to 24 cm, depending on the image being obtained. Collected images and video loops were saved on the Ibex Pro at the time of sampling. Gravidity was determined on the basis of the presence or absence of embryos. Images and frozen video stills were then used to measure (via proprietary software preinstalled on the Ibex EVO II ultrasound) pup diameter (centimeters) along the transverse axis.

### Tagging and tracking

The dorsal fin of gravid sharks was affixed with either Smart Position and Temperature Transmitting tags (SPOT6 tag, Wildlife Computers; tiger shark) or the KiwiSat (model K2F 176F, Lotek Inc.; scalloped hammerhead). Fin-mounted tags were coated with antifouling materials to minimize biofouling and affixed to the first dorsal fin. Geographic location of each tagged shark was determined via Doppler-shift calculation made by the ARGOS Data Collection and Location Service (www.argos-system.org) whenever the shark’s tag broke the water’s surface and transmitted. Location accuracy was dependent on the number of tag transmissions received by ARGOS satellites. ARGOS provides location accuracy using LCs 3, 2, 1, 0, A, B, and Z (in decreasing accuracy), corresponding with the following error estimates: LC3 < 250 m, 250 m < LC2 < 500 m, and 500 m < LC1 < 1500 m. The error estimates associated with LCs A and B are reported to be >1 and >5 km, respectively. LC Z estimates are inaccurate or unreliable and were removed from the dataset before any analysis. Because of irregular surfacing of sharks (and thus irregular transmission rates and variation in satellite coverage at any given time), positional data were interpolated and regularized using a Bayesian state-space model that also accounts for ARGOS satellite telemetry precision. These position data were interpolated and regularized to daily estimates in the R statistical software using the package foieGras (*34*). Tracks were then mapped with positions color-coded by date, in ArcGIS Pro (Esri).

## Notes

View/request a protocol for this paper from *Bio-protocol*.
